# Verum- versus Sham-Acupuncture on Alzheimer's Disease (AD) in Animal Models: A Preclinical Systematic Review and Meta-Analysis

**DOI:** 10.1155/2020/5901573

**Published:** 2020-03-31

**Authors:** Fei-Yi Zhao, Qiang-Qiang Fu, Zhen Zheng, Li-Xing Lao, Hua-Ling Song, Zumin Shi

**Affiliations:** ^1^Department of Nursing, School of International Medical Technology, Shanghai Sanda University, Shanghai, China; ^2^Yangpu Hospital, Tongji University School of Medicine, Shanghai, China; ^3^School of Health and Biomedical Sciences, RMIT University, Bundoora, Victoria 3083, Australia; ^4^School of Chinese Medicine, The University of Hong Kong, 10 Sassoon Road, Pokfulam, Hong Kong; ^5^Virginia University of Integrative Medicine, Fairfax, VA 22031, USA; ^6^School of Public Health, Shanghai University of Traditional Chinese Medicine, Shanghai, China; ^7^Human Nutrition Department, College of Health Science, QU Health, Qatar University, Doha, Qatar

## Abstract

**Background:**

Alzheimer's disease (AD) is a common health condition affecting senile people and leads to severe cognitive dysfunctions. Acupuncture has been shown to be a possible alternative natural remedy for AD in some animal studies.

**Objective:**

To perform a systematic review to identify the effect of verum-acupuncture compared with sham-acupuncture on learning and memory performance among animal models of AD.

**Methods:**

Experimental animal studies of treating AD via verum- and sham- acupuncture were searched in nine electronic databases, including Sciverse ScienceDirect, PubMed, Springer, Ebsco Medline, AMED, EMBASE (Elsevier), Scopus (Elsevier), PsycINFO (ProQuest), and OVID from the dates of the databases' inception to June 2019. The Morris water maze test was considered as an outcome measure. The software Revman 5.3 and Stata 16.0 were used to conduct the meta-analysis. Heterogeneity was examined by using I^2^ statistics. The publication bias was assessed via Begg's test by Stata 16.0.

**Results:**

Twelve studies involving 229 animals met the inclusion criteria. Most of the studies had a moderate quality according to SYRCLE's risk of bias tool for animal studies. The results of the meta-analysis indicated that verum-acupuncture could reduce the escape latency (MD = −12.90, 95% CI (−17.08, −8.71), *p* < 0.001) and increase the time spent in the original platform quadrant (MD = 7.28, 95% CI (4.23, 10.33), *p* < 0.001) and frequency of the crossing former platform (MD = 2.01, 95% CI (1.53, 2.50), *p* < 0.001) compared with the sham-acupuncture.

**Conclusions:**

Acupuncture is effective in improving cognitive functions in AD animal models, and this benefit is more than just a placebo effect. Further clinical trials are needed to confirm the findings.

## 1. Background and Introduction

Alzheimer's disease (AD) is one of the most important causes of dementia [1], contributing to more than 60% of all cases [2, 3]. As a progressive neurodegenerative disorder without efficient therapeutic agents, AD irreversibly and progressively impairs memory, language, thinking, and other critical mental functions [4, 5]. A report from WHO estimated that dementia contributed 11.2% of years spent living with a disability in those aged 60 years and above, which is more than cancer, cardiac vascular disease, and stroke [6]. Disability and dependence due to AD then further place a burden on caregivers [7]. Because of a high prevalence, AD has an enormous socioeconomic impact. In 2017, an estimated 18.4 billion hours and valued at over $232 billion of care was provided to patients of AD or other dementias by over 16 million family members and other unpaid caregivers [8]; in 2018, approximately $277 billion was paid for health care, long-term care, and hospice services for the senior over 65-year-old with dementia [8]. The number of cases was even predicted to affect 1 in 85 individuals worldwide by 2050 [9]. Furthermore, the countries/regions with the greatest number of affected individuals are China and the developing western Pacific, Western Europe, and the US [10]. With the globally speedy increase in the aging population, AD represents one of the great public health challenges of the 21st century [11] and deserves much more attention.

No ideal treatment for AD is available today [12]. Though some interventions including currently prevailing medication regimens are likely to improve symptoms temporarily, it cannot stop or reverse the progression of AD either [12]. Cholinesterase inhibitors (ChEIs) are commonly used in the clinical practice. Donepezil, galantamine, and rivastigmine are a class of medications that are currently approved by the US Food and Drug Administration (FDA) for treating patients in the mild to moderate stage of AD [13, 14]. FDA has also approved a daily use of 23 mg donepezil compound to treat the symptoms of moderate to severe AD [15]. Nevertheless, raised costs and increased risk of serious adverse events linked to ChEIs treatment has been challenged [16]. For instance, adverse reactions including insomnia, nausea, and diarrhea may already occur with just 10 mg/d of donepezil [17], let alone the incidence of rivastigmine-induced adverse events found even higher than that of donepezil [18]. Memantine is another FDA-approved medication for moderate to severe AD but is also limited to the increased risk for a variety of adverse effects such as somnolence, hypertension, weight gain, and confusion [19].

Due to the acute health risk of AD and the limitations of pharmacotherapy, there have been multiple anecdotal reports and controlled studies over the past decade to examine the effectiveness of acupuncture on AD. Acupuncture is an integral part of traditional Chinese medicine with considerable clinical efficacy and minimal side effects [20] that can be traced back for at least 4000 years [21]. It is a technique of skin being inserted and penetrated with the thin, solid, and metallic needles that are manipulated by the hands (manual acupuncture, MA) or by electrical stimulation (electroacupuncture, EA). The entire TCM model for the action of acupuncture is that balanced *Yin* and *Yang* and *Qi*, along with *blood* and *body fluid* is vital to optimal health, whereas any imbalance or interruption of the homeostasis would result in illness and diseases [21]. In many East Asian countries including China, Japan, Korea, as well as some regions in the US and Europe, acupuncture is popular and has been extensively used for multiple neurodegenerative diseases including AD [12], while the therapeutic effect is inconclusive. A systematic review published in 2009 implied insufficient evidence to prove acupuncture was effective on both cognitive function and activities of daily living (ADL) among patients with AD [22]. However, another systematic meta-analysis published in 2015 suggested that acupuncture was not only more effective than drugs but also might further enhance the effects of drugs in improving both cognitive function and ADL [23]. Additionally, there is still hot debate whether acupuncture is merely a psychological or “placebo” effect, accompanied with both positive and negative reports published again and again [24–26].

Animal study is often used to examine the efficacy of acupuncture as the factors that might interfere with the results can be well controlled in the study. Findings from some animal studies have shown that acupuncture might be beneficial for AD [27–29]. However, there has been no systematic review concerning acupuncture treatment on AD animals. In fact, a systematic review based on animal data might inform the planning and promote the likelihood of success of future clinical trials, preclude unnecessary study replication, identify what is valuable in further research, and elucidate the underling mechanism of acupuncture [30]. Thus, we aimed to evaluate the effectiveness of verum-acupuncture compared with sham-acupuncture on cognitive dysfunctions in AD animals. We choose studies with sham-acupuncture as the control because it is a better way to address the psychological or “placebo” effects of acupuncture.

## 2. Materials and Methods

We formulated the research question “Is the verum-acupuncture more likely to attenuate the cognitive dysfunction in AD animals when compared to sham-acupuncture therapy?” This guiding question was defined by the PICO strategy. In order to address this question, we investigated and assessed AD animals (P = participants) treated with verum-acupuncture therapy (I = intervention) and compared with those AD animals receiving sham-acupuncture therapy (C = intervention comparison or control), aiming at verifying the changes in learning and memory functions reflected by Morris water maze (O = outcome).

### 2.1. Search Strategy and Eligibility Criteria

We searched the following nine English electronic databases: Sciverse ScienceDirect, PubMed, Springer, Ebsco Medline, AMED, EMBASE (Elsevier), Scopus (Elsevier), PsycINFO (ProQuest), and OVID in June 2019, using the combined terms “(electroacupuncture, acupuncture, meridian, or acupoint) and (dementia or Alzheimer).” The retrieval was conducted with no restrictions regarding the year, but the language was limited to English. We also tried to identify additional studies from other source, including the reference list of the included papers and those grey literatures such as conference papers and unpublished papers. Two independent reviewers were responsible for the literatures searching work and quality evaluation. The detailed search strategy is shown in [Supplementary-material supplementary-material-1].

### 2.2. Inclusion and Exclusion Criteria

#### 2.2.1. Inclusion Criteria

1. P: AD model animals
2. I: the acupuncture therapy was limited to only MA and EA
3. C: intervention in the control group was limited to only sham-acupuncture therapy
4. O: the cognitive performance of AD animals was reflected by results of the Morris water maze test (outcome indicators such as escape latency, time spent in the quadrant in which the former platform was located, frequency of crossing through the former platform, and swimming speed)
5. Language: only studies published in English
6. No publication date limit was set, and the literature retrieval work was conducted in June 2019

#### 2.2.2. Exclusion Criteria

1. The acupuncture therapy was laser acupuncture, auricular acupuncture, or other acupuncture techniques
2. Studies that only compared different acupuncture techniques/acupoints or only compared the different intervention frequencies within the same acupuncture technique
3. Studies that only compared acupuncture with TCM/western medication or only compared a combotherapy of acupuncture and TCM/western medication with either acupuncture or TCM/western medication
4. Studies that had no control group or the intervention in the control group was wait-list control (AD animal without any therapy) or western medication control
5. Duplicate articles (if multiple literature reports were judged to be the same experiment, the one with the largest sample size and the most comprehensive information was retained)

### 2.3. Data Extraction

EndNote software (Version X7) was used to store the results of the searches and to remove duplicates. One researcher (ZFY) generated the search strategy, searched the potential databases, and drew up a list of all the records. Two evaluators (ZFY and FQQ) independently evaluated and screened the articles according to the inclusion and exclusion criteria. Disagreements were solved together through discussion with a third reviewer (SHL). Two reviewers (ZFY and FQQ) independently extracted the data and proofread the information.

The following data were extracted: the last name of the first author, publication year, the species, gender, age, weight range, and numbers of the included animals, modeling methodology of AD, approach of treatment with timing and frequency in the verum-acupuncture and control groups, the acupoints used, subtest indicators selected in the Morris water maze test, the results of each study (positive or negative), and the underlying mechanism of acupuncture. The final outcomes were extracted if several outcomes were presented. When the outcome data were only shown graphically, we attempted to contact the authors to obtain the original and detailed data; if we received no response to our request, GetData Graph Digitizer software (Version 2.25) was used to measure the data.

### 2.4. Risk of Bias Assessment

A ten-item checklist introduced by “SYRCLE's risk of bias tool for animal studies” [31] was used to assess the study quality. The quality of each study was evaluated by the judgment of “Yes,” “No,” or “Unclear.” “Yes” judgment indicated a low risk of bias; “No” judgment indicated high risk of bias; “Unclear” judgment was suggested if insufficient details had been reported to evaluate the risk of bias properly. Each study could score ranging from zero to ten, with a high score represented a superior quality. The assessment was done by two independent reviewers (ZFY and FQQ), and disagreements were solved through consensus-oriented discussion or by consulting a third researcher (SHL).

## 3. Statistical Analysis

Meta-analysis was performed by Revman software (Version 5.3) to assess the effectiveness of the therapy. As the major outcomes were continuous variables, the mean ± standard deviation (mean ± SD) was calculated with 95% confidence interval (CI). Level of heterogeneity across the studies was tested using the *Q*-test and *I*^2^ test. The results were pooled using a fixed effects model when the *p* value was >0.10 in the *Q*-test and the *I*^2^ value was ≤50%. Otherwise, a random effects model was applied. When significant heterogeneity existed, the subgroup analysis was further conducted to measure the pooled effect and to explore the source of heterogeneity based on animal species, acupuncture methods, and modeling methods. Sensitivity analyses and metaregressions were adopted to explore the source of heterogeneity if possible as well. The publication bias was assessed via Egger's test and Begg's test by Stata software (Version 16.0).

## 4. Results

**Figure 1:**
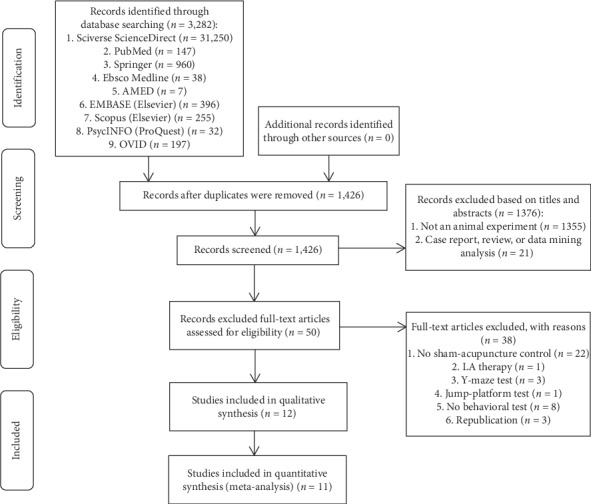
Flow diagram of the study selection process.

3282 possibly relevant studies were identified in the initial search. After removing the duplicates, we screened the titles and the abstracts of 1426 remaining records, and 1376 records were excluded. For further screening, 50 full-text articles were downloaded. Eventually, 12 studies met the inclusion criteria. The selection process flow diagram is shown in Figure 1.

### 4.1. Study Characteristics

Characteristics of all the 12 included studies are listed in Table 1.

#### 4.1.1. Species, Characteristics, and Features of the Animal

The 12 studies included 229 AD model animals, 115 of which were in an acupuncture group and 114 of which were in a sham-acupuncture group. Among the 229 animals, there were 189 males and 20 females, while 1 study involved a total of 20 animals but did not indicate the gender of them [28].

Of the 12 studies, rats were used in four studies, including SD rats used in two studies [29, 38] and Wistar rats used in two studies [34, 35]. The remaining studies adopted mice for modeling, including three studies adopted APP/PS1 double transgenic mice [28, 33, 40], and the rest used SAMP8 mice.

Nearly, all 12 studies mentioned the age of the experimental animals except for one study [40]. In mice-related studies, the age of mice ranged from 3 to 12 months; in rats-related studies, the age range was from 2 to 5 months. Meanwhile, 6 studies mentioned the weight of the experimental animals [29, 33–35, 38, 40]. The weight ranges were 20–27 grams in mice models and 220–350 grams in rat models.

#### 4.1.2. Model Preparation Methods

Different approaches were used to establish an AD animal model in rats and mice. In the four rats models, intraperitoneal injection of scopolamine (SCO) was used to establish an AD model in one study [29] and intracerebroventricular (ICV) injection of A*β*_1–42_ was used in two studies [34, 35], and the ICV injection of A*β*_1–40_ was used in the rest one study [38]. In mice models, three studies adopted APP/PS1 double transgenic mice model [28, 33, 39], and the rest used SAMP8 mice model as mentioned above [27, 32, 36, 37, 40].

#### 4.1.3. Acupoints Selections

Acupoints selection is one of the critical factors affecting the efficacy during acupuncture practice [41].

The frequency of acupoints used in the 12 included studies was sorted out and listed as follows (Table 2). Furthermore, the selection of acupoints is arranged in descending order of times and frequency of use. As can be seen, the most frequently used acupoint was GV20. In view of the basic theory of TCM, due the special location of GV20, which is on the vertex of the head where all the *Yang meridians* meet [42], stimulating GV20 could clear the mind and improve mental function [43]. In addition, CV6, CV12, CV17, SP10, and ST36 were used as a set of acupoints in five included studies to strengthen the foundation of Qi. Other used acupoints included BL23 and TE4.

### 4.2. Study Quality Evaluation

The quality assessments of 12 included papers are illustrated in Tables 3 and 4, and the score of the study quality ranged from 2 to 8 out of a total 10 points.

Among the 12 studies, only two studies described the specific random method [37, 39]. Similarly, only two studies clearly showed that the baseline characteristics had been evaluated or adjusted among the groups before the intervention [35, 39], and two studies reported that caregivers or investigators were blinded during the study [29, 33], respectively. No study clarified if allocation was adequately concealed and what randomization technique was applied in the trial. Expected outcomes were comprehensively reported in all the studies, and no study was identified with other problems that could result in high risk of bias.

### 4.3. Outcomes Analyses of Morris Water Maze

Among the 12 studies, one study [40] adopted and measured total travelled distance, line crossover number, and frequency of entries to the IL zone as the subtest outcomes of Morris water maze, which were different from other studies. Therefore, this study was only included for descriptive analysis, while the remaining studies were included for a meta-analysis, and the results were illustrated as follows.

#### 4.3.1. Escape Latency

**Figure 2:**
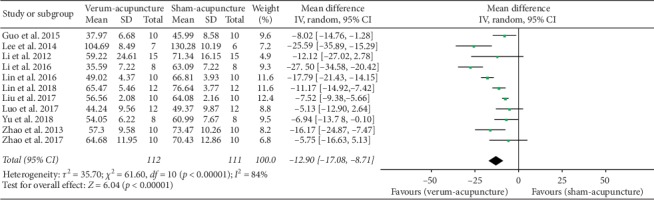
Forest plot of verum- versus sham-acupuncture in escape latency.

Eleven studies [27–29, 32–39] adopted escape latency as an outcome indicator, and all reported a positive effect of verum-acupuncture in reducing escape latency. However, there was a high heterogeneity among the studies (*p* < 0.00001, *I*^2^ = 84%), and random effects model thereby was performed for analysis. According to the results, there was a significant difference between verum-acupuncture and sham-acupuncture in reducing escape latency (MD = −12.90, 95% CI (−17.08, −8.71), *p* < 0.00001) (Figure 2).

Due to the heterogeneity, we further conducted subgroup analysis according to animal species (mice or rats), modeling methods (SAMP8 and APP/PS1, intraperitoneally injected with SCO, ICV A*β*_1–42_, or ICV A*β*_1–40_), and different acupuncture therapies (MA or EA). The results are showed as follows (Table 5).

 
*Subgroup Analyses of Escape Latency*
(a) Animal species: compared with sham - acupuncture, verum-acupuncture could reduce the escape latency in both mice (MD = −11.03, 95% CI (−15.31, −6.74), *p* < 0.00001) and rats (MD = −16.74, 95% CI (−27.74, −5.75), *p*=0.003). Neither the mice (*I*^2^ = 79%) nor the rats (*I*^2^ = 88%) observed a striking decline in heterogeneity through this subgroup analysis.
(b) Acupuncture methods: compared with sham-acupuncture, both verum-MA and verum-EA could decrease the escape latency (MD = –12.85, 95% CI (–20.63, –5.07), *p*=0.001 in MA; MD = –12.96, 95% CI (–18.29, –7.63), *p* < 0.001 in EA). The subgroup analysis found that MA had mildly reduced heterogeneity (*I*^2^ = 66%), while EA did not (*I*^2^ = 90%).
(c) Modeling methods: compared with sham-acupuncture, verum-acupuncture significantly shortened the escape latency in SAMP8 (MD = −9.51, 95% CI (−15.28, −3.73), *p*=0.001), APP/PS1 (MD = −12.03, 95% CI (−18.19, −5.86), *p*=0.0001), intraperitoneally injected with SCO (MD = −25.59, 95% CI (−35.88, −15.29), *p* < 0.00001), ICV A*β*_1–40_ (MD = −8.03, 95% CI (−14.77, −1.28), *p*=0.02), but not in ICV A*β*_1–42_ (MD = −17.21, 95% CI (−37.35, 2.94), *p*=0.09). No subgroup analysis based on modeling methods reduced heterogeneity except for SAMP8 (*I*^2^ = 26%).

#### 4.3.2. Platform Crossover Numbers

**Figure 3:**
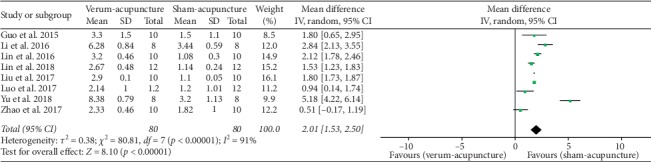
Forest plot of verum- versus sham-acupuncture in platform crossover numbers.

Platform crossover numbers were adopted as an outcome in eight studies [27, 28, 33–35, 37–39], and all of these studies reported the positive effect of verum-acupuncture in increasing platform crossover numbers. However, there was a heterogeneity among the studies (*p* < 0.00001, *I*^2^ = 91%) and random effects model thereby was used for analysis. A significant difference was found between verum- and sham-acupuncture in increasing platform crossover numbers (MD = 2.01, 95% CI (1.53, 2.50), *p* < 0.00001) (Figure 3).

Because of the high heterogeneity, we further conducted subgroup analysis according to animal species (mice or rats), modeling methods (SAMP8, APP/PS1, ICV A*β*_1–42_, or ICV A*β*_1–40_), and different acupuncture therapies (MA or EA). The results are illustrated as follows (Table 6).

 
*Subgroup Analyses of Platform Crossover Numbers*

- Animal species: compared with sham-acupuncture, verum-acupuncture increased the platform crossover numbers in both mice (MD = 1.53, 95% CI (1.16, 1.89), *p* < 0.00001) and rats (MD = 3.29, 95%CI (1.47, 5.11), *p*=0.0004). The subgroup analysis observed that mice had slightly reduced heterogeneity (*I*^2^ = 84%), while rats did not (*I*^2^ = 91%).
- Acupuncture methods: compared with sham-acupuncture, verum-acupuncture showed more elevation in platform crossover numbers in both MA (MD = 0.69, 95% CI (0.17, 1.21), *p*=0.009) and EA (MD = 2.40, 95% CI (1.87, 2.94), *p* < 0.00001). The subgroup analysis found that MA had absolutely eliminate heterogeneity (*I*^2^ = 0%), while EA further increased heterogeneity (*I*^2^ = 92%).
- Modeling methods: Compared with sham-acupuncture, verum-acupuncture had a more striking effect in increasing platform crossover numbers in SAMP8 (MD = 0.69, 95% CI (0.17, 1.21), *p*=0.009), APP/PS1 (MD = 1.81, 95% CI (1.56, 2.05), *p* < 0.00001), ICV A*β*_1–40_ (MD = 1.80, 95% CI (0.65, 2.95), *p*=0.002), and ICV A*β*_1–42_ (MD = 3.99, 95% CI (1.70, 6.29), *p*=0.0007). The subgroup analysis observed that both SAMP8 (*I*^2^ = 0%) and APP/PS1 (*I*^2^ = 70%) had reduced heterogeneity, while ICV A*β*_1–42_ did not (*I*^2^ = 93%).

#### 4.3.3. Time Spent in the Original Platform Quadrant

**Figure 4:**
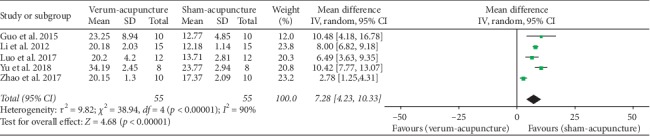
Forest plot of verum- versus sham-acupuncture in time spent in the original platform quadrant.

Five studies [27, 32, 34, 37, 38] adopted time spent in the original platform quadrant as an outcome, and all of these studies reported a significantly better effect of verum-acupuncture than sham-acupuncture. Random effects model was used for analysis due to the high heterogeneity (*p* < 0.00001, *I*^2^ = 90%). As suggested, there was a remarkable difference between verum- and sham-acupuncture (MD = 7.28, 95% CI (4.23, 10.33), *p* < 0.00001) (Figure 4).

Due to the heterogeneity, we further conducted subgroup analysis according to animal species (mice or rats), modeling methods (SAMP8, ICV A*β*_1–42_, or ICV A*β*_1–40_), and different acupuncture therapies (MA or EA). The results are indicated in Table 7.

*Subgroup Analyses of Time Spent in the Original Platform Quadrant*.

- Animal species: compared with sham-acupuncture, verum-acupuncture evidently increased time spent in the original platform quadrant in both mice (MD = 5.74, 95% CI (2.06, 9.43, *p* = 0.002) and rats (MD = 10.43, 95% CI (7.98, 12.87), *p* < 0.00001). The subgroup analysis observed that rats had no heterogeneity (*I*^2^ = 0%), while mice had a high heterogeneity (*I*^2^ = 93%).
- Acupuncture methods: compared with sham-acupuncture, verum-acupuncture had a more remarkable effect in prolonging time spent in the original platform quadrant in both MA (MD = 5.74, 95% CI (2.06, 9.43, *p* = 0.002) and EA (MD = 10.43, 95% CI (7.98, 12.87), *p* < 0.00001). No heterogeneity (*I*^2^ = 0%) was found in EA, whereas marked heterogeneity (*I*^2^ = 93%) was observed in MA.
- Modeling methods: compared with sham-acupuncture, verum-acupuncture noticeably extended time spent in the original platform quadrant in SAMP8 (MD = 5.74, 95% CI (2.06, 9.43, *p* = 0.002), ICV A*β*_1–40_ (MD = 10.48, 95% CI (4.18, 16.79), *p* = 0.001), and ICV A*β*_1–42_ (MD = 10.42, 95% CI (7.76, 13.07), *p* < 0.00001). Heterogeneity could not be reduced via the subgroup analysis of modeling methods.

#### 4.3.4. Swimming Speed

**Figure 5:**
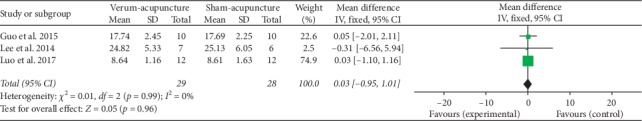
Forest plot of verum- versus sham-acupuncture in swimming speed.

Three studies [29, 37, 38] adopted swimming speed as an outcome. No statistical heterogeneity was found among each study (*p*=0.99, *I*^2^ = 0%), and fixed effects model thereby was therefore used for analysis. Our data suggested that there was no significant difference in swimming speed between receiving verum-acupuncture and receiving sham-acupuncture (MD = 0.03, 95% CI (–0.95, 1.01), *p*=0.96) (Figure 5).

#### 4.3.5. Other Indicators

In addition to the outcomes mentioned above, there were some other indicators of the Morris water maze test also mentioned in the included studies (Table 5). Limited to the quantity and importance, they seemed to be not suitable for a meta-analysis but could be synthesized with descriptive analysis.

As shown in Table 8, the percentage of time spent in platform quadrant was reported in 2 studies [29, 39] and was found to be increased in verum-acupuncture. Similarly, search path length [28] and first time of crossing the platform [35] were reduced in verum-acupuncture, respectively. Number of line crossing, number of entries to the IL zone, and total distance travelled were mentioned in only one study [40]. Compared with sham-acupuncture, those three outcomes were increased in verum-acupuncture [40]. All of the above results implied the effective improvements of acupuncture on cognitive performances among AD animals.

### 4.4. Sensitivity Analysis

To investigate the sources of heterogeneity, we performed sensitivity analysis based on the outcome of escape latency. We chose influence analysis of sensitivity analysis. This process, in which one study was removed at a time, was conducted to recalculate the combined estimate on the remaining studies and evaluate the stability of the results. We did not conduct sensitivity analysis for the other three measures of MWM because of the small number of studies (<10).

**Figure 6:**
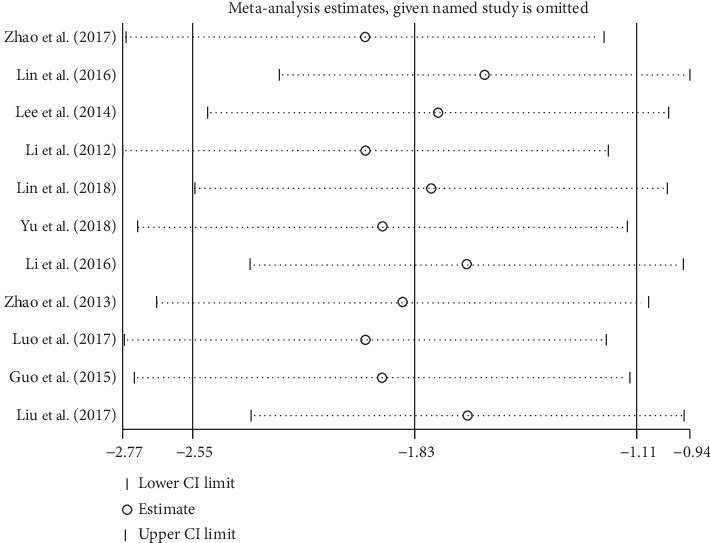
Sensitivity analysis on the outcome of escape latency.

Figure 6 shows the results of sensitivity analysis. After eliminating 11 studies one by one, the effect of the remaining studies was within the 95% CI of the total effect.

### 4.5. Metaregression Analysis

Using escape latency as the outcome measure, we conducted univariate metaregressions to explore the sources of heterogeneity by treating publication year, study sample size, animal age, animal species (rats and mice), acupuncture methods (MA and EA), and acupuncture duration as covariates. Meanwhile, multifactor metaregressions were conducted to find the sources of heterogeneity by taking modeling methods (SAMP8 and APP/PS1, intraperitoneally injected with SCO, ICV A*β*_1–42_, or ICV A*β*_1–40_) and acupoints selections as covariates. We did not conduct metaregression according to the animal weight due to the lack of large amounts of data, even though we tried to contact the authors of included papers.

In metaregression, heterogeneity of escape latency could not be substantially explained by publication year (*I*^2^ = 80.88%, *τ*^2^ = 37.97, *p*=0.195), study sample size (*I*^2^ = 86.85%, *τ*^2^ = 35.24, *p*=0.134), animal age (*I*^2^ = 77.40%, *τ*^2^ = 36.41, *p*=0.122), animal species (*I*^2^ = 83.44%, *τ*^2^ = 44.41, *p*=0.282), acupuncture methods (*I*^2^ = 85.23%, *τ*^2^ = 50.98, *p*=0.976), acupuncture duration (*I*^2^ = 84.95%, *τ*^2^ = 50.92, *p*=0.696), modeling methods (*I*^2^ = 86.85%, *τ*^2^ = 48.72, *p*=0.5093), and acupoints selections (*I*^2^ = 85.98%, *τ*^2^ = 47.83, *p*=0.4730). These covariates were irrelevant to heterogeneity (Supplemental Fig. [Supplementary-material supplementary-material-1]–[Supplementary-material supplementary-material-1]).

### 4.6. Publication Bias Test

**Figure 7:**
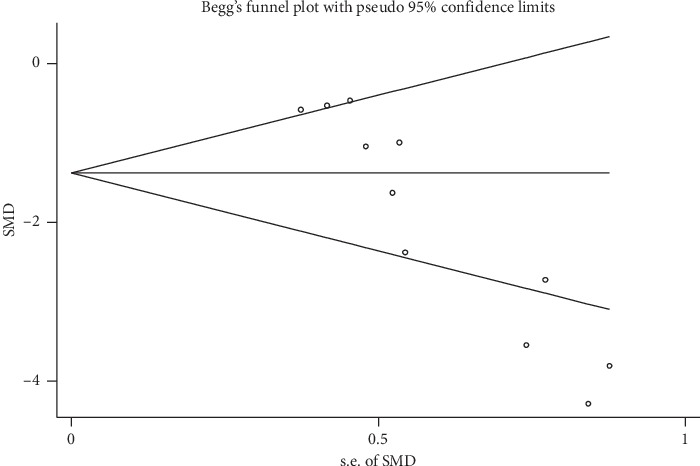
Begg's publication bias test of escape latency.

We conducted a publication bias test for the outcome of escape latency using Egger's test (Pr > |*z*| = 0.001 < 0.05, continuity corrected) and Begg's test (Pr > |*z*| = 0.001 < 0.05, continuity corrected, Figure 7). The potential existence of publication bias was revealed. We did not conduct a publication bias test for the other outcome measures because of the small number of studies (<10).

### 4.7. Possible and Proposed Mechanisms

The major signaling pathways were also investigated to gain a better understanding of the possible and potential mechanisms of acupuncture-induced cognitive improvement (Table 1).

Several signaling pathways associated with the neuroprotective mechanisms of acupuncture were extensively investigated in those studies, including correction of the abnormal cerebral glycolysis metabolism [36, 39], enhancement of synaptic plasticity [34, 35, 38], antiapoptosis and repair of neuronal cells [27, 38], degradation and decreases of A*β* protein deposits [34, 39], upregulation of the hippocampal expression of BDNF [28, 29, 33] and TrkB [33], and reduction of neuron loss and apoptosis [32].

Acupuncture therapy was also shown to attenuate the AD-induced cognitive impairments via upregulating the stabilisation of the cellular signal [37] but downregulating GSK-3*β* [34] or negative regulation of neuroinflammation [40].

## 5. Discussion

### 5.1. Summary of Findings

To the best of our current knowledge, it is the first systematic metaanalysis investigating the effectiveness of verum-acupuncture on AD animal models compared with sham-acupuncture with the results of the Morris water maze test as the outcome assessment.

We found low to moderate level of evidence that verum-acupuncture had a superior effect in reducing escape latency, increasing platform crossover numbers, and time spent in the original platform quadrant when compared with sham-acupuncture. However, no significant difference in swimming speed between two therapies was observed. The findings suggest that acupuncture may have a neuroprotective effect in animal models of AD, and this benefit is more than just a placebo effect.

### 5.2. Comparison with Other Reviews

Park et al. published the first review on the effect of acupuncture on AD in animal-based research in 2017 [44]. According to Park's review, MA and EA appeared to generate similar benefits to AD rodents when targeted the same acupoints [44]. One limitation of Park's review is that it included two studies without any experiment and assessment for animal behaviors. Furthermore, most of the studies Park et al. included only involved a comparison between acupuncture and wait-list control. The lack of an effective placebo- or sham-acupuncture control reduces the validity of the results. No meta-analysis was included in that review as well [44]. Our subgroup analysis suggests that the effect of EA seemed slightly better than MA. This is likely because we included more persuasive studies in the review.

### 5.3. Sources of Heterogeneities

The high heterogeneities were found among the studies. We thereby conducted the subgroup analysis to identify the source of heterogeneities. Subgroup classification was based on animal species, modeling methods, and acupuncture methods. It is encouraging to note that, after subgroup analysis, effects of acupuncture on AD animals remained positive, and heterogeneity was partially well-explained. For instance, verum-MA could increase platform crossover numbers in SAMP8 mice models compared with sham-MA (MD = 0.69, 95% CI (0.17, 1.21), *p*=0.009, *I*^2^ = 0%). However, the heterogeneities among the studies based on escape latency and time spent in the original platform quadrant could not be completely clarified despite further sensitivity analyses and metaregression analyses.

### 5.4. Potential Mechanisms

The underlying mechanisms linking acupuncture and the treatment of AD were partially summarized in our result section. In addition to the proposed mechanisms listed in Table 1, we identified three relevant review papers, including one systematic review without meta-analysis [45] and two narrative reviews [44, 46]. According to the review by Leung et al. [45] and Cao et al. [46], acupuncture could alleviate cognitive impairments by modulating multiple signaling pathways involved in neuroprotection and neurorepair. For instance, promotion of cholinergic neural transmission is one of the main mechanisms of acupuncture, including alleviation of the loss of hippocampal acetylcholinesterase (AChE) immunoreactivity [45, 47] and elevation of the activity of hippocampal choline acetyltransferase (ChAT) [45, 48]. Similarly, acupuncture increases activity of hippocampal triosephosphate isomerase (TPI) [36], deficiency of which may suppress the process of glycolysis and eventually leads to degeneration of cerebral functions [46, 49]. Both inhibition of oxidative stress and attenuation of neuronal apoptosis contribute the therapeutic mechanisms of acupuncture as well [45, 46]. Acupuncture primarily suppress the oxidative stress and the subsequent cell death triggered by oxidative damage via minimizing the damages of neuronal mitochondria in the hippocampus [45, 46]. A significant decline of dying neurons was detected in the hippocampus of AD rodent undergoing real-acupuncture compared with the sham-acupuncture control [46, 50].

### 5.5. Challenge and Implications

The results from our study appear to be promising, and it is however too early to draw any definitive and positive conclusion. There are several challenges impacting on translating preclinical evidence into clinical evidence.

Firstly, the results of our systematic review show that acupuncture has the potential to improve cognitive impairments in AD, and this finding is consistent with results of a few clinical trials, which also report that acupuncture improves quality of life of AD patients [51–53]. The therapeutic effect was enhanced by combined acupuncture and oral administration of conventional medication such as huperzine A capsules [52]. However, methodological problems from both clinical trials and animal experiments impact on the strength of evidence. More randomized clinical trials with better study designs thereby are needed.

Secondly, in view of Table 1, acupuncture is found to attenuate cerebral injuries through multiple mechanisms. Details of the critical mechanisms closely associated with cognitive functions are insufficient at present. For instance, it is unclear how acupuncture improves on systemic- and neuroinflammation, oxidative stress, and synaptic plasticity among AD models. Once biomarkers are identified, they can be used in future clinical studies.

Thirdly, the acupuncture protocols used in the included studies are heterogenous. Some studies applied a set of acupoints [27, 32, 36, 37, 40], while others stimulated one acupoint only [28, 33, 39]; three studies intervened for 28 days [28, 33, 38], while other six studies only took half of the time [27, 29, 32, 34, 35, 40]. The following questions remained to be answered: Does the selection of acupoints (more or less) or/and the intervention duration directly affect the effects of acupuncture on AD-induced cognitive dysfunction? How big is this effect? After all, how to organize the least number of acupoints to achieve the best therapeutic effect is of great value to reduce the cost of clinical practice.

Finally, clinically, acupuncture is applied based on the basic theory of TCM, which emphasizes the concept of “syndrome types” [54]. “Treatment based on syndrome differentiation” (TBSD) is the core principle of TCM; that is, patients are classified into different TCM syndrome types according to their clinical signs and symptoms and then corresponding treatments are prescribed [54, 55]. Briefly, AD patients with different syndrome types are usually given different acupuncture prescriptions in clinical practice [56, 57]. Unfortunately, in the studies we retrieved, TBSD was usually ignored even though the AD animal modeling methods were varied. Establishing different AD models based on syndrome types, and then verifying the efficacy of acupuncture prescriptions in these models, seems to be more in line with the laws of TCM research and might make the findings from the preclinical research more relevant to clinical practice.

## 6. Limitations

Our systematic review and meta-analysis have several limitations. Firstly, the total number of studies and the total sample size were relatively small. Secondly, we did not include other acupuncture techniques such as laser and auricular acupuncture. Thirdly, the quality of included studies was unsatisfactory. Among the included researches, one study scored points in seven items [39], four studies in six items [29, 33, 35, 37], and the reminding studies only in five items according to ‘SYRCLE's bias tool [31]. The reasons for the low quality of most studies were mainly due to (1) unclear states of baseline comparability and randomization methods of allocation and (2) absence of blinding methods and random selection for outcome evaluation. Our results also indicated that there was a publication bias. Further rigorous and well-designed animal studies with larger sample sizes are required.

## 7. Conclusions

Acupuncture might be effective in ameliorating cognitive dysfunction in AD animals, and this benefit is more than just a placebo effect. Furthermore, our findings implies that the neuroprotective effects of acupuncture might be achieved through blocking the apoptosis and loss of neuronal cells, correcting of the abnormal cerebral glycolysis metabolism, enhancing of synaptic plasticity, degrading and decreasing of A*β* deposits, and upregulating of the hippocampal expression of BDNF. However, these findings should be interpreted cautiously due to the limitations of the included studies.

## Figures and Tables

**Table 1:** Study characteristics of 12 included studies.

Author	Species (M/F)	Age (month)	Weight (g)	Model	Animals and study groups/group size	Acupuncture interventions	Acupoints	Sham-acupuncture prescription	MWM outcomes	MA/EA compared with other interventions (no treatment = AD model)	Potential mechanisms
Zhao et al. [27]	(i) SAMP8 mice (50/0) (ii) SAMR_1_ mice (10/0)	8	NR	SAMP8	(i) Control: SAMR_1_/*n* = 10 (ii) SAMP8/*n* = 10 (iii) SAMP8 + sham-NSCs transplantation (NSCs-T)/*n* = 10 (iv) SAMP8 + NSCs-T/*n* = 10 (v) SAMP8 + NSCs-T + MA/*n* = 10 (vi) SAMP8 + NSCs-T + sham-MA/*n* = 10	MA, 210 s/d for 15 d (suspended only on day 7)	CV6, CV12, CV17, SP10, ST36	(i) MA on fixed nonacupoints (ii) The acupuncture method, frequency, and duration are the same as those of the verum-acupuncture group	(i) Escape latency (ii) Platform crossover number (iii) Time spent in the original platform quadrant	(i) Compared with no treatment, *p* < 0.05 (ii) Compared with sham-NSCs-T, *p* < 0.05 (iii) Compared with NSCs-T, *p* < 0.05 (iv) Compared with NSCs-T + sham-MA, *p* < 0.05 ^*∗*^(i)–(iv): compariso*n* for “escape latency” (v) Compared with no treatment, *p* < 0.05 (vi) Compared with sham-NSCs-T, *p* < 0.05 (vii) Compared with NSCs-T, *p* < 0.05 (viii) Compared with NSCs-T + sham-MA, *p* < 0.05 ^*∗*^(v)–(viii): comparison for “platform crossover number” ^*∗*^(v)–(viii): compariso*n* for “platform crossover number” (ix) Compared with no treatment, *p* < 0.05 (x) Compared with sham-NSCs-T, *p* < 0.05 (xi) Compared with NSCs-T, *p* < 0.05 (xii) Compared with NSCs-T + sham-MA, *p* < 0.05 ^*∗*^(ix)–(xii): compariso*n* for “time spent in the original platform quadrant”	(i) MA upregulates expression of bFGF, EGF, and BDNF (ii) MA upregulates expression of bFGF, EGF, BDNF, BMP_4_, SDF_1_, and VEGF mRNA (iii) MA promotes proliferation and differentiation of NSCs

Lin et al. [28]	(i) APP/PS1 mice (30, sex NR) (ii) Wild-type mice (10, sex NR)	3	NR	APP/PS1	(i) Control: wild-type/*n* = 10 (ii) APP/PS1/*n* = 10 (iii) APP/PS1 + EA/*n* = 10 (iv) APP/PS1 + sham-EA/*n* = 10	EA, 30 min/d for 4 weeks	GV20	(i) EA on a nonacupoint (ii) The acupuncture method, frequency, and duration are the same as those of the verum-acupuncture group	(i) Escape latency (ii) Platform crossover number (iii) Search path length	(i) Compared with no treatment, *p* < 0.01 (ii) Compared with no treatment, *p* < 0.01 (iii) Compared with no treatment, *p* < 0.01	(i) EA reduces overexpression of A*β*_1–42_ and inhibits neuronal apoptosis in the hippocampus (ii) EA increases expression levels of BDNF and proBDNF in the hippocampus

Lee et al. [29]	SD rats (33/0)	2	220–240	Intraperitoneally injected with scopolamine (SCO)	(i) Control: saline/*n* = 7 (ii) SCO/*n* = 7 (iii) SCO + MA GV20/*n* = 7 (iv) SCO + MA TE4/*n* = 6 (v) SCO + sham-MA/*n* = 6	MA, 5 min/d for 14 d	GV20 and TE4	(i) MA on fixed nonacupoint (ii) The acupuncture method, frequency, and duration are the same as those of the verum-acupuncture group	(i) Escape latency (ii) Swimming speed (iii) Percentage of time spent in the original platform quadrant	(i) MA GV20 compared with no treatment, *p* < 0.05 (ii) MA TE4 compared with no treatment, *p* > 0.05 ^*∗*^(i)-(ii): compariso*n* for “escape latency” (iii) MA GV20 compared with no treatment, *p* < 0.05 (iv) MA TE4 compared with no treatment, *p* > 0.05 ^*∗*^(iii)-(iv): compariso*n* for “swimming speed” (v) MA GV20 compared with no treatment, *p* < 0.05 (vi) MA TE4 compared with no treatment, *p* > 0.05 ^*∗*^(v)-(vi): compariso*n* for “percentage of time spent in the original platform quadrant”	(i) MA GV20 increases the levels of ChAT, BDNF, and CREB proteins in the hippocampus (ii) MA GV20 increases the expression of CHT_1_, VAChT, BDNF, and CREB mRNA in the hippocampus
Li et al. [32]	(i) SAMP8 mice (45/0) (ii) SAMR_1_ mice (15/0)	7.5	NR	SAMP8	(i) Control: SAMR_1_/*n* = 15 (ii) SAMP8/*n* = 15 (iii) SAMP8 + MA/*n* = 15 (iv) SAMP8 + sham-MA/*n* = 15	MA, 210 s/d for 15 d (suspended only on day 8)	CV6, CV12, CV17, SP10, ST36	(i) MA on fixed nonacupoints (ii) The acupuncture method, frequency, and duration are the same as those of the verum-acupuncture group	(i) Escape latency (ii) Time spent in the original platform quadrant	(i) Compared with no treatment, *p* < 0.05 (ii) Compared with sham-MA, *p* < 0.05 ^*∗*^(i)-(ii): compariso*n* for “escape latency” (iii) Compared with no treatment, *p* < 0.05 (iv) Compared with sham-MA, *p* < 0.05 ^*∗*^(iii)-(iv): compariso*n* for “time spent in the original platform quadrant”	(i) MA reduces neuron loss in the hippocampal regions CA3 and dentate gyrus

Lin et al. [33]	(i) APP/PS1 (36/0) (ii) Wild-type mice (12/0)	12	25 ± 2	APP/PS1	(i) Control: wild-type/*n* = 12 (ii) APP/PS1/*n* = 12 (iii) APP/PS1 + EA/*n* = 12 (iv) APP/PS1 + sham-EA/*n* = 12	EA, 30 min/d for 4 weeks	GV20	(i) EA on a nonacupoint (ii) The acupuncture method, frequency, and duration are the same as those of the verum-acupuncture group	(i) Escape latency (ii) Platform crossover number	(i) Compared with no treatment, *p* < 0.05 (ii) Compared with sham-EA, *p* < 0.05 ^*∗*^(i)-(ii): compariso*n* for “escape latency” (iii) Compared with no treatment, *p* < 0.05 (iv) Compared with sham-EA, *p* < 0.05 ^*∗*^(iii)-(iv): comparison for ‘platform crossover number'	(i) EA improves *N*-acetylaspartate, glutamate, and myoinositol metabolism (ii) EA decreases neuronal cells destruction (iii) EA increases expression of BDNF and TrkB
Yu et al. [34]	Wistar rat (56/0)	4-5	310 ± 20	ICV injection of A*β*_1–42_	(i) Control: no procedure/*n* = 8 (ii) ICV injection of saline/*n* = 8 (iii) ICV injection of A*β*_1–42_/*n* = 8 (iv) ICV injection of A*β*_1–42_ + sham-EA/*n* = 8 (v) ICV injection of A*β*_1–42_ + EA 2 Hz/*n* = 8 (vi) ICV injection of A*β*_1–42_ + EA 30 Hz/*n* = 8 (vii) ICV injection of A*β*_1–42_ + EA 50 Hz/*n* = 8	EA, 20 min/d for 15 d (suspended only on day 8)	BL23, GV20	(i) Only the surfaces of GV20 and BL23 were stimulated, but the needles were inserted without current (ii) The acupuncture method, frequency, and duration are the same as those of the verum-acupuncture group	(i) Escape latency (ii) Platform crossover number (iii) Time spent in the original platform quadrant	(i) Compared with no treatment, *p* < 0.01 (ii) Compared with sham-EA, *p* < 0.01 (iii) EA 50 Hz/EA 30 Hz compared with EA 2 Hz, *p* < 0.05 ^*∗*^(i)–(iii): compariso*n* for “escape latency” (iv) Compared with no treatment, *p* < 0.01 (v) Compared with sham-EA, *p* < 0.01 (vi) EA 50 Hz compared with EA 2 Hz/EA 30 Hz *p* < 0.05 ^*∗*^(iv)–(vi): comparison for “platform crossover number” (vii) Compared with no treatment *p* < 0.01 (viii) EA 50 Hz/EA 30 Hz compared with sham-EA *p* < 0.01 (xi) EA 50 Hz compared with EA 2 Hz/EA 30 Hz *p* < 0.05 ^*∗*^(vii)–(ix): compariso*n* for “time spent in the original platform quadrant”	(i) EA increased synaptic curvatures, decreased the width of synaptic clefts, and thickened postsynaptic densities (ii) EA decreases the expression of GSK-3β, amyloid precursor protein, and A*β*_1–40_

Li et al. [35]	Wistar rat (48/0)	4-5	300–350	ICV injection of A*β*_1–42_	(i) Control: no procedure/*n* = 8 (ii) ICV injection of saline/*n* = 8 (iii) ICV injection of A*β*_1–42_/*n* = 8 (iv) ICV injection of A*β*_1–42_ + sham-EA/*n* = 8 (v) ICV injection of A*β*_1–42_ + EA 2 Hz/*n* = 8 (vi) ICV injection of A*β*_1–42_ + EA 50 Hz/*n* = 8	EA, 20 min/d for 15 d (suspended only on day 8)	BL23, GV20	(i) Only the surfaces of GV20 and BL23 were stimulated, but the needles were inserted without current (ii) The acupuncture method, frequency, and duration are the same as those of the verum-acupuncture group	(i) Escape latency (ii) Platform crossover number (iii) First time of crossing the original platform	(i) Compared with no treatment *p* < 0.01 (ii) Compared with sham-EA *p* < 0.01 ^*∗*^(i)–(iii): compariso*n* for “escape latency” (iii) EA 50 Hz compared with EA 2 Hz *p* < 0.05 (iv) Compared with no treatment *p* < 0.01 (v) Compared with sham-EA *p* < 0.01 (vi) EA 50 Hz compared with EA 2 Hz *p* < 0.05 ^*∗*^(iv)–(vi): comparison for “platform crossover number” (vii) Compared with no treatment *p* < 0.01 (viii) Compared with sham-EA *p* < 0.01 (ix) EA 50 Hz compared with EA 2 Hz *p* < 0.05 ^*∗*^(vii)–(ix): comparison for “first time of crossing the original platform”	(i) EA enhances hippocampal synaptic transmission

Zhao L [36]	(i) SAMP8 mice (30/0) (ii) SAMR_1_ mice (10/0)	8	NR	SAMP8	(i) Control: SAMR_1_/*n* = 10 (ii) SAMP8/*n* = 10 (iii) SAMP8 + MA/*n* = 10 (iv) SAMP8 + sham-MA/*n* = 10	MA, 210 s/d for 18 d	CV6, CV12, CV17, SP10, ST36	(i) MA on fixed nonacupoints (ii) The acupuncture method, frequency, and duration are the same as those of the verum-acupuncture group	(i) Escape latency	(i) Compared with no treatment *p* < 0.01 (ii) Compared with sham-MA *p* < 0.01	(i) MA increases TPI activity
Luo et al. [37]	(i) SAMP8 mice (36/0) (ii) SAMR_1_ mice (12/0)	8	NR	SAMP8	(i) Control: SAMR_1_/*n* = 12 (ii) SAMP8/*n* = 12 (iii) SAMP8 + MA/*n* = 12 (iv) SAMP8 + sham-MA/*n* = 12	MA, 210 s/d for 18 d	CV6, CV12, CV17, SP10, ST36	(i) MA on fixed nonacupoints (ii) The acupuncture method, frequency, and duration are the same as those of the verum-acupuncture group	(i) Escape latency (ii) Platform crossover number (iii) Time spent in the original platform quadrant (iv) Swimming speed	(i) Compared with no treatment *p* < 0.05 (ii) Compared with sham-MA *p* < 0.05 ^*∗*^(i)-(ii): comparison for “escape latency” (iii) Compared with no treatment *p* < 0.01 (iv) Compared with sham-MA *p* < 0.01 ^*∗*^(iii)-(iv): comparison for “platform crossover number” (v) Compared with no treatment *p* < 0.01 (vi) Compared with sham-MA *p* < 0.01 ^*∗*^(v)-(vi): comparison for “time spent in the original platform quadrant” (vii) Compared with no treatment *p* < 0.05 (viii) Compared with sham-MA *p* > 0.05 ^*∗*^(vii)-(viii): comparison for “swimming speed”	(i) MA upregulates G-protein activity and stabilisation of the cellular signal

Guo et al. [38]	SD rats (40/0)	2-3	250–300	ICV injection of A*β*_1–40_	(i) ICV injection of saline/*n* = 10 (ii) ICV injection of A*β*_1–40_/*n* = 10 (iii) ICV injection of A*β*_1–40_ + EA/*n* = 10 (iv) ICV injection of A*β*_1–40_ + sham-EA/*n* = 10	EA, 30 min/d for 24 d	BL23, GV20	(i) MA on fixed nonacupoints (ii) The acupuncture method, frequency, and duration are the same as those of the verum-acupuncture group	(i) Escape latency (ii) Platform crossover number (iii) Time spent in the original platform quadrant (iv) Swimming speed	(i) Compared with no treatment *p* < 0.05 (ii) Compared with sham-EA *p* < 0.05 ^*∗*^(i)-(ii): comparison for “escape latency” (iii) Compared with no treatment *p* < 0.05 (iv) Compared with sham-EA *p* < 0.05 ^*∗*^(iii)-(iv): comparison for “platform crossover number” (v) compared with no treatment *p* < 0.01 (vi) Compared with sham-EA *p* < 0.01 ^*∗*^(v)-(vi): comparison for “time spent in the original platform quadrant” (vii) Compared with no treatment *p* < 0.05 (viii) Compared with sham-EA *p* > 0.05 ^*∗*^(vii)-(viii): comparison for “swimming speed”	(i) EA reduces the neuronal apoptosis in the hippocampus (ii) EA promotes the recovery of synaptic function (iii) EA decreases the expression of Notch1 and Hes1 mRNA in the hippocampus

Liu et al. [39]	(i) APP/PS1 (0/30) (ii) Wild-type mice (0/10)	9	NR	APP/PS1	(i) Control: wild-type/*n* = 10 (ii) APP/PS1/*n* = 10 (iii) APP/PS1 + EA/*n* = 10 (iv) APP/PS1 + sham-EA/*n* = 10	EA, 30 min/d, 5 d/week for 4 weeks	GV20	(i) EA on a nonacupoint (ii) The acupuncture method, frequency, and duration are the same as those of the verum-acupuncture group	(i) Escape latency (ii) Platform crossover number (iii) Percentage of time spent in the original platform quadrant	(i) Compared with no treatment, *p* < 0.01 (ii) Compared with sham-EA *p* < 0.01 ^*∗*^(i)-(ii): comparison for “escape latency” (iii) Compared with no treatment *p* < 0.01 (iv) Compared with sham-EA *p* < 0.01 ^*∗*^(iii)-(iv): comparison for “platform crossover number” (v) Compared with no treatment *p* < 0.01 (vi) Compared with sham-EA *p* < 0.01 ^*∗*^(v)-(vi): comparison for “percentage of time spent in the original platform quadrant”	(i) EA increases brain glucose metabolism (ii) EA increases expression of GLUT1 and GLUT3 in the hippocampus and cortex (iii) EA reduces A*β*_1–42_ deposition (iv) EA increases phosphorylation of AMPK, AKT, and mTOR in the hippocampus and cortex

Li et al. [40]	(i) SAMP8 mice (9/0) (ii) SAMR_1_ mice (3/0)	NR	20–25	SAMP8	(i) Control: SAMR_1_/*n* = 3 (ii) SAMP8/*n* = 3 (iii) SAMP8 + MA/*n* = 3 (iv) SAMP8 + sham-MA/*n* = 3	MA, 15 min/d for 15 d (suspended only on day 8)	CV6, CV12, CV17, SP10, ST36	(i) MA on fixed nonacupoints (ii) The acupuncture method, frequency, and duration are the same as those of the verum-acupuncture group	(i) Total distance travelled (ii) Number of line crossover (iii) Number of entries to the IL zone	(i) Compared with no treatment *p* < 0.05 (ii) Compared with no treatment *p* < 0.05 (iii) Compared with no treatment *p* < 0.05	(i) MA reduces hippocampal inflammation and neuron nuclear damage (ii) MA downregulates PI3K/PDK1/nPKC/Rac 1 signaling pathway

*Abbreviations.* NR, no record; SD, Sprague Dawley; M, male; F, female; MA, manual acupuncture; EA, electroacupuncture; SAMP8, senescence-accelerated mouse prone 8; SAMR_1_, senescence-accelerated mouse resistance 1; MWM, Morris water maze; A*β*, amyloid *β*-peptide; ICV, intracerebroventricular; bFGF, basic fibroblast growth factor; EGF, epidermal growth factor; BDNF, brain-derived neurotrophic factor; BMP_4_, bone morphogenic protein 4; SDF_1_, stromal cell-derived factor 1; VEGF, vascular endothelial growth factor; AMPK, adenosine monophosphate-activated protein kinase; ChAT, choline acetyltransferase; CREB, cAMP-response element-binding protein; CHT_1_, choline transporter 1; VAChT, vesicular acetylcholine transporter; TrkB, tropomyosin receptor kinase B; GSK-3β, glycogen synthase kinase-3*β*; TPI, triose phosphate isomerase; mTOR, mammalian target of rapamycin; BL23, Shenshu; CV6, Qihai; CV12, Zhongwan; CV17, Danzhong; GV20, Baihui; SP10, Xuehai; ST36, Zusanli; TE4, Yangji.

**Table 2:** Frequency of acupoints used in studies.

Frequency, *n* (%)	Acupoints	Involved studies
7 (58.33)	GV20	[28, 29, 33–35, 38, 39]
5 (41.67)	CV6	[27, 32, 36, 37, 40]
	CV12	
	CV17	
	SP10	
	ST36	
3 (25.00)	BL23	[34, 35, 38]
1 (8.33)	TE4	[29]

*Abbreviations.* BL23, Shenshu; CV6, Qihai; CV12, Zhongwan; CV17, Danzhong; GV20, Baihui; SP10, Xuehai; ST36, Zusanli; TE4, Yangji.

**Table 3:** Methodological quality assessment of the included studies.

Study	1	2	3	4	5	6	7	8	9	10	Scores
Zhao et al. [27]	U	N	U	Y	U	U	Y	Y	Y	Y	5
Lin et al. [28]	U	N	U	Y	U	N	Y	Y	Y	Y	5
Lee et al. [29]	U	N	U	Y	Y	U	Y	Y	Y	Y	6
Li et al. [32]	U	N	U	Y	U	N	Y	Y	Y	Y	5
Lin et al. [33]	U	N	U	Y	Y	U	Y	Y	Y	Y	6
Yu et al. [34]	U	N	U	Y	U	U	Y	Y	Y	Y	5
Li et al. [35]	U	Y	U	Y	U	N	Y	Y	Y	Y	6
Zhao et al. [36]	U	N	U	Y	U	N	Y	Y	Y	Y	5
Luo et al. [37]	Y	N	U	Y	U	N	Y	Y	Y	Y	6
Guo et al. [38]	U	N	U	Y	U	U	Y	Y	Y	Y	5
Liu et al. [39]	Y	Y	U	Y	U	U	Y	Y	Y	Y	7
Li et al. [40]	U	N	U	Y	U	N	Y	Y	Y	Y	5

*Notes.* (1) Generation of animal allocation sequence was random; (2) each group was similar or was at adjusted at baseline; (3) the allocation was adequately concealed; (4) animals were housed at random; (5) both animal breeders and researchers were blinded for the intervention of each animal received; (6) animals were selected randomly for outcome evaluation; (7) outcome evaluator was blinded; (8) the incomplete outcome data were absolutely addressed; (9) reports of the research were free of selective outcome reporting; (10) study was evidently free of other potential issues which may cause bias. Y, yes (low-risk bias); N, no (high risk bias); U, unclear.

**Table 4:** Methodological quality assessment of the included studies.

Study quality scores	Studies	*n* (%)
7	[40]	1 (8.33)
6	[29, 33, 35, 37]	4 (33.33)
5	[27, 28, 32, 34, 36, 38, 40]	7 (58.33)

**Table 5:** Subgroup analyses of escape latency.

Subgroup	MD	LL	HL	Df	*I* ^2^	*Z*	*p*
Animal species							
Mice	−11.03	−15.31	−6.74	6	79	5.04	<0.00001
Rats	−16.74	−27.74	−5.75	3	88	2.98	0.003
Acupuncture methods							
MA	−12.85	−20.63	−5.07	4	66	3.24	0.001
EA	−12.96	−18.29	−7.63	5	90	4.77	<0.00001
Modeling							
SAMP8	−9.51	−15.28	−3.73	3	26	3.23	0.001
ICV A*β*_1–40_	−8.03	−14.77	−1.28	—	—	2.33	0.02
ICV A*β*_1–42_	−17.21	−37.35	2.94	1	94	1.67	0.09
APP/PS1	−12.03	−18.19	−5.86	2	92	3.82	0.0001
Intraperitoneally injected with SCO	−25.59	−35.88	−15.29	—	—	4.87	<0.00001

*Abbreviations.* MA, manual acupuncture; EA, electroacupuncture; ICV, intracerebroventricular.

**Table 6:** Subgroup analyses of platform crossover numbers.

Subgroup	MD	LL	HL	Df	*I* ^2^	*Z*	*p*
Animal species							
Mice	1.53	1.16	1.89	4	84	8.24	<0.00001
Rats	3.29	1.47	5.11	2	91	3.55	0.0004
Acupuncture methods							
MA	0.69	0.17	1.21	1	0	2.61	0.009
EA	2.40	1.87	2.94	5	92	8.83	<0.00001
Modeling methods							
SAMP8	0.69	0.17	1.21	1	0	2.61	0.009
ICV A*β*_1–40_	1.80	0.65	2.95	—	—	3.06	0.002
ICV A*β*_1–42_	3.99	1.70	6.29	1	93	3.41	0.0007
APP/PS1	1.81	1.56	2.05	2	70	14.25	<0.00001

*Abbreviations*. MA, manual acupuncture; EA, electroacupuncture.

**Table 7:** Subgroup analyses of time spent in the original platform quadrant.

Subgroup	MD	LL	HL	Df	*I* ^2^	*Z*	*p*
Animal species							
Mice	5.74	2.06	9.43	2	93	3.05	0.002
Rats	10.43	7.98	12.87	1	0	8.36	<0.00001
Acupuncture methods							
MA	5.74	2.06	9.43	2	93	3.05	0.002
EA	10.43	7.98	12.87	1	0	8.36	<0.00001
Modeling methods							
SAMP8	5.74	2.06	9.43	2	93	3.05	0.002
ICV A*β*_1–40_	10.48	4.18	16.79	—	—	3.26	0.001
ICV A*β*_1–42_	10.42	7.76	13.07	—	—	7.70	<0.00001

*Abbreviations.* MA, manual acupuncture; EA, electroacupuncture; ICV, intracerebroventricular.

**Table 8:** Other outcomes of the Morris water maze.

Other outcomes of the Morris water maze	Included studies	Compared with the sham-acupuncture group
Percentage of time spent in platform quadrant	[29, 39]	+
Search path length	[28]	−
First time of crossing the platform	[35]	−
Number of line crossing	[40]	+
Number of entries to the IL zone	[40]	+
Total distance travelled	[40]	+

## Data Availability

The data used to support the findings of this study are available from the corresponding author upon request.

## Supplementary Materials

Supplementary Materials Appendix 1: search strategy The following search strategy was used for PubMed, and this search strategy was also suitable for other electronic databases. Appendix 2: figures of meta-regression. Supplemental Figure 1: univariate meta-regression based on publication year as covariate Supplemental Figure 2: univariate meta-regression based on study sample size as covariate Supplemental Figure 3: univariate meta-regression based on animal age as covariate Supplemental Figure 4: univariate meta-regression based on animal species size as covariate Supplemental Figure 5: univariate meta-regression based on acupuncture methods size as covariate Supplemental Figure 6: univariate meta-regression based on acupuncture duration size as covariate.
