# Supplementary material for: Verum- versus Sham-Acupuncture on Alzheimer's Disease (AD) in Animal Models: A Preclinical Systematic Review and Meta-Analysis
Source: Biomed Res Int. 2020 Mar 31;2020:5901573. doi: 10.1155/2020/5901573 (PMC7150729; doi:10.1155/2020/5901573)

Appendix 1. Search strategy

The following search strategy was used for PubMed, and this search strategy was also suitable for other electronic databases:

**Number**	**Search terms**
1	dementia
2	Alzheimer disease
3	Alzheimer's disease
4	cognitive disorders
5	cognitive impairment
6	dement*
7	Alzheimer*
8	1 OR 2-7
9	acupuncture therapy
10	acupuncture
11	acupoints
12	acupunct*
13	electroacupuncture
14	electro-acupuncture
15	ear acupuncture
16	auricular acupuncture
17	scalp acupuncture
18	body acupuncture
19	elongated needle
20	9 OR 10-19
21	Sham acupuncture
22	Sham-acupuncture
23	Placebo acupuncture
24	Placebo-acupuncture
25	21 OR 22-24
26	Morris water maze
27	MWM
28	behavioral test
29	26 OR 27-28
30	8 AND 20 AND 25 AND 29

Appendix 2. Figures of meta-regression

Supplemental Fig.1 Univariate meta-regression based on publication year as covariate

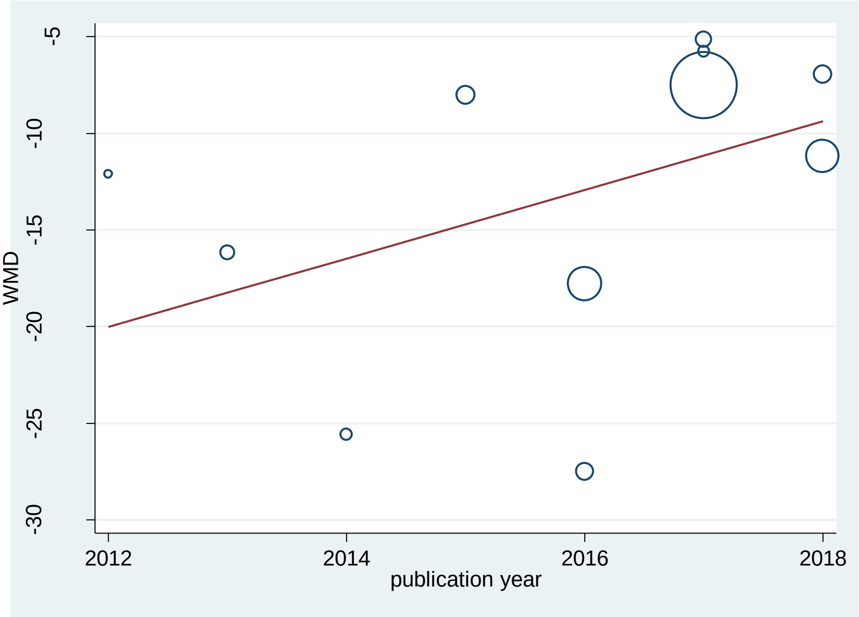

Supplemental Fig.2 Univariate meta-regression based on study sample size as covariate

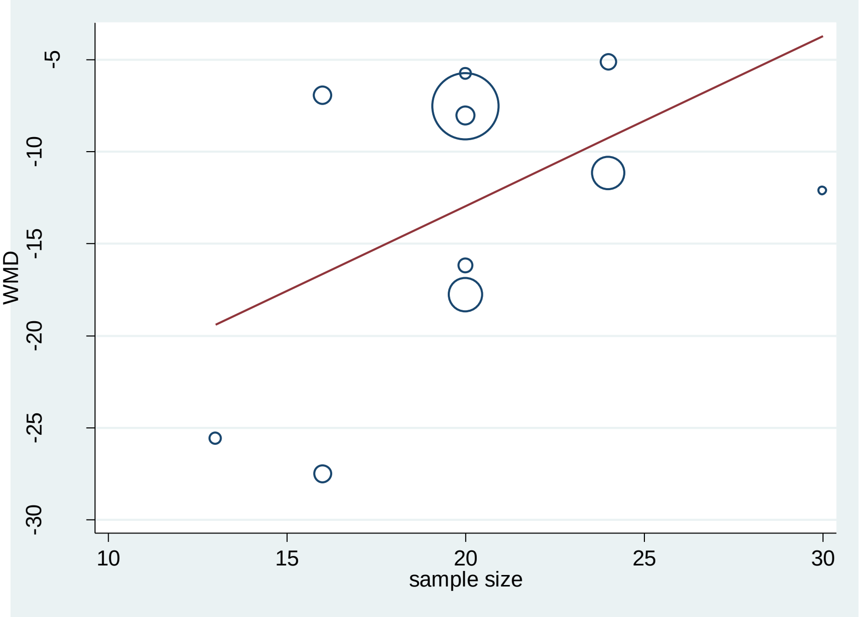

Supplemental Fig.3 Univariate meta-regression based on animal age as covariate

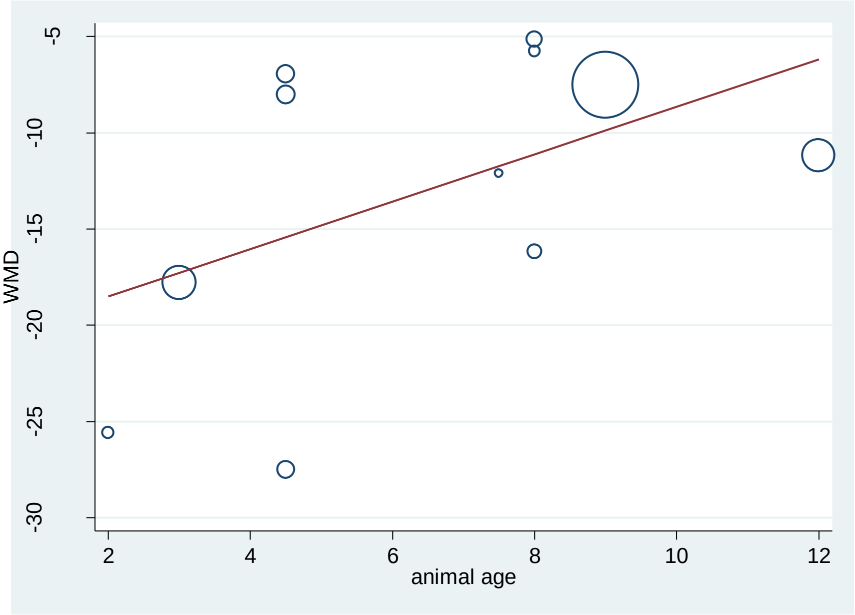

Supplemental Fig.4 Univariate meta-regression based on animal species size as covariate

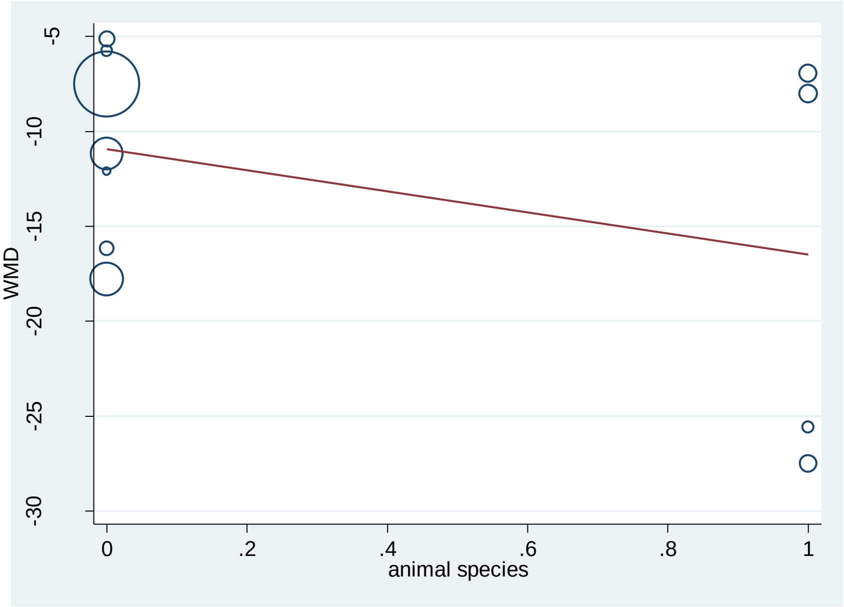

Supplemental Fig.5 Univariate meta-regression based on acupuncture methods size as covariate

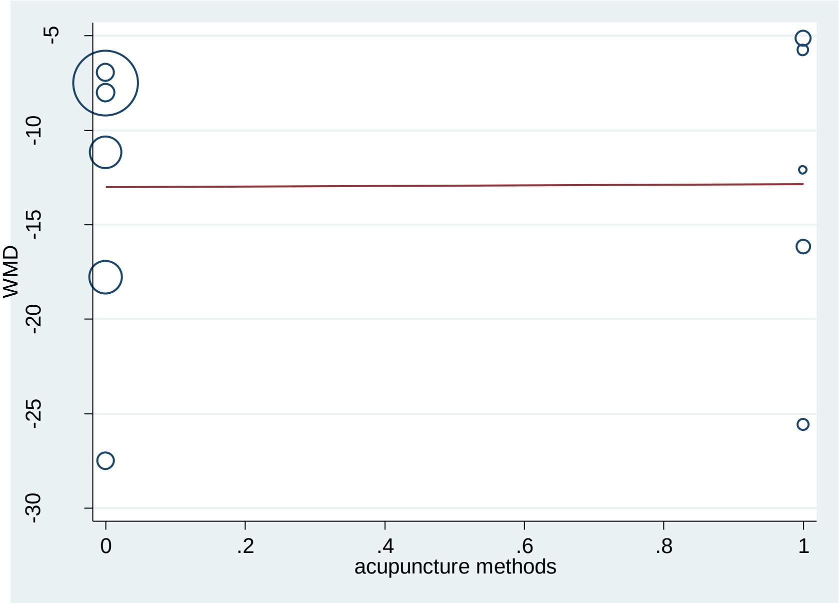

Supplemental Fig.6 Univariate meta-regression based on acupuncture duration size as covariate

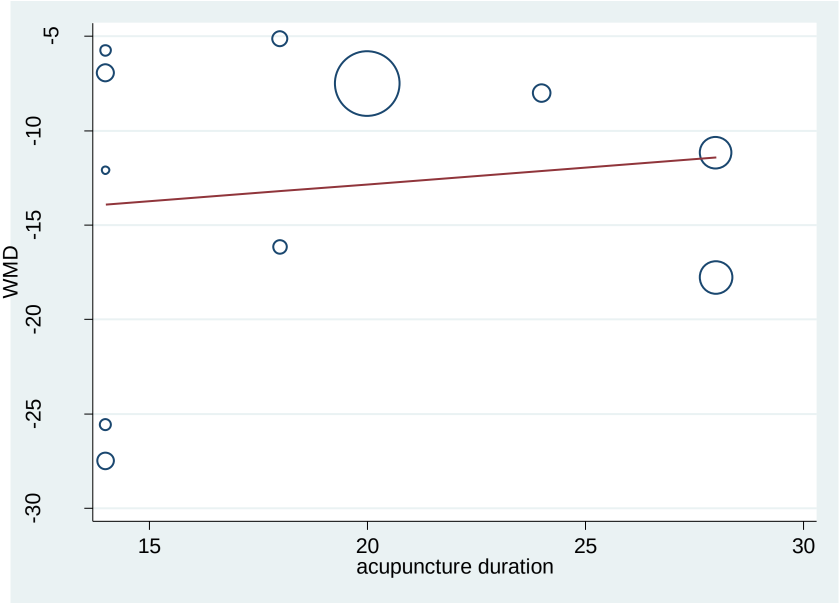

Supplement: Supplementary Materials — Appendix 1: search strategy The following search strategy was used for PubMed, and this search strategy was also suitable for other electronic databases. Appendix 2: figures of meta-regression. Supplemental Figure 1: univariate meta-regression based on publication year as covariate Supplemental Figure 2: univariate meta-regression based on study sample size as covariate Supplemental Figure 3: univariate meta-regression based on animal age as covariate Supplemental Figure 4: univariate meta-regression based on animal species size as covariate Supplemental Figure 5: univariate meta-regression based on acupuncture methods size as covariate Supplemental Figure 6: univariate meta-regression based on acupuncture duration size as covariate. [file 5901573.f1.pdf]
